# Identification and Characterization of FGF2-Dependent mRNA: microRNA Networks During Lens Fiber Cell Differentiation

**DOI:** 10.1534/g3.113.008698

**Published:** 2013-10-18

**Authors:** Louise Wolf, Chun S. Gao, Karen Gueta, Qing Xie, Tiphaine Chevallier, Nikhil R. Podduturi, Jian Sun, Ivan Conte, Peggy S. Zelenka, Ruth Ashery-Padan, Jiri Zavadil, Ales Cvekl

**Affiliations:** * Department of Genetics, Albert Einstein College of Medicine, Bronx, New York 10461; † Department of Ophthalmology and Visual Sciences, Albert Einstein College of Medicine, Bronx, New York 10461; ‡ Laboratory of Molecular and Developmental Biology, National Eye Institute (NEI), Bethesda, Maryland 20892; § Sackler School of Medicine and Sagol School of Neuroscience, Tel-Aviv University, 69978 Ramat Aviv, Tel Aviv, Israel; ** Department of Pathology and New York University Center for Health Informatics and Bioinformatics, New York University Langone Medical Center, New York, New York 10000; †† Telethon Institute of Genetics and Medicine, Via Pietro Castellino 111, I-80131 Naples, Italy

**Keywords:** c-Maf, Dicer1, differentiation, FGF2, lens, microRNAs, signaling

## Abstract

MicroRNAs (miRNAs) and fibroblast growth factor (FGF) signaling regulate a wide range of cellular functions, including cell specification, proliferation, migration, differentiation, and survival. In lens, both these systems control lens fiber cell differentiation; however, a possible link between these processes remains to be examined. Herein, the functional requirement for miRNAs in differentiating lens fiber cells was demonstrated via conditional inactivation of Dicer1 in mouse (*Mus musculus*) lens. To dissect the miRNA-dependent pathways during lens differentiation, we used a rat (*Rattus norvegicus*) lens epithelial explant system, induced by FGF2 to differentiate, followed by mRNA and miRNA expression profiling. Transcriptome and miRNome analysis identified extensive FGF2-regulated cellular responses that were both independent and dependent on miRNAs. We identified 131 FGF2-regulated miRNAs. Seventy-six of these miRNAs had at least two *in silico* predicted and inversely regulated target mRNAs. Genes modulated by the greatest number of FGF-regulated miRNAs include DNA-binding transcription factors Nfib, Nfat5/OREBP, c-Maf, Ets1, and N-Myc. Activated FGF signaling influenced bone morphogenetic factor/transforming growth factor-β, Notch, and Wnt signaling cascades implicated earlier in lens differentiation. Specific miRNA:mRNA interaction networks were predicted for c-Maf, N-Myc, and Nfib (DNA-binding transcription factors); Cnot6, Cpsf6, Dicer1, and Tnrc6b (RNA to miRNA processing); and Ash1l, Med1/PBP, and Kdm5b/Jarid1b/Plu1 (chromatin remodeling). Three miRNAs, including miR-143, miR-155, and miR-301a, down-regulated expression of c-Maf in the 3′-UTR luciferase reporter assays. These present studies demonstrate for the first time global impact of activated FGF signaling in lens cell culture system and predicted novel gene regulatory networks connected by multiple miRNAs that regulate lens differentiation.

Cellular differentiation is a tightly controlled process in which a single or multiple extracellular signals regulate a multitude of processes, including exit from cell cycle followed by cellular differentiation. Seven signal transduction pathways—hedgehog, Janus Kinase/signal transducer and activator of transcription, Notch, nuclear receptors, receptor tyrosine kinase, transforming growth factor-β (TGF-β), and Wnt—control the vast majority of differentiation processes. In the nucleus, these signals reach the transcriptional apparatus and result in regulation of specific target genes by signal-regulated transcription factors (Barolo and Posakony 2002). Specificity of signaling is controlled at multiple levels by a variety of mechanisms (Barolo and Posakony 2002; Firth and Baker 2009; Turner and Grose 2010). Spatially controlled expression and/or gradients of concentration of individual growth factors, their cofactors and/or inhibitors, and receptors provide regulation at the level of ligand−receptor interaction. In the cytoplasmic compartment, signals can be relayed via alternate pathways and amplified via posttranslational modifications, including phosphorylation/dephosphorylation, of specific proteins residing within the multiprotein transitional signaling complexes (Ramos 2008; Roskoski 2012). When the signal reaches the nucleus, specificity of signaling is ensured through the cooperative interactions between multiple DNA-binding transcription factors, often regulated by different signaling pathways, and insufficiency of individual factors (Barolo and Posakony 2002). Recent studies on regulation of gene expression identified novel posttranscriptional mechanisms through microRNAs (miRNAs) with a number of miRNAs exhibiting tissue-specific or tissue-preferred patterns of expression (Bartel 2004; Pauli *et al.* 2011; Conte *et al.* 2013). High-throughput detection of both RNAs and miRNAs by oligonucleotide arrays, quantitative polymerase chain reaction (qPCR), and/or by massively parallel sequencing allow modeling of genetic networks that control key cellular processes, including terminal differentiation (Ivey and Srivastava 2010; Pauli *et al.* 2011).

Ocular lens is a unique model for differentiation studies because the lens is composed of a single type of cell that reaches different stages of differentiation, either as lens fibers or lens epithelium depending on its spatial localization in the lens (Lovicu and McAvoy 2005). Lens development and differentiation are regulated by bone morphogenetic factor (BMP)/TGF-β, fibroblast growth factor (FGF), Notch, and Wnt signaling (Lovicu and McAvoy 2005; Smith *et al.* 2010; Gunhaga 2011). FGF/mitogen-activated protein kinase (MAPK) signaling (Dailey *et al.* 2005; Lovicu and McAvoy 2005; Robinson 2006; Lanner and Rossant 2010; Turner and Grose 2010) is required for the formation of lens progenitor cells from the common preplacodal progenitor cell population (Streit 2004, 2007) via regulation of Pax6 function. Inactivation of Ndst1, an enzyme from heparin sulfate biosynthetic pathway that cooperates with FGF signaling, prevented the formation of lens and retina (Pan *et al.* 2006; Qu *et al.* 2011). The inactivation of three FGF receptors (FGFR1, 2, and 3) disrupted cell-cycle exit and multiple aspects of the lens fiber cell differentiation (Garcia *et al.* 2005; Zhao *et al.* 2008). FGF signaling is also needed for survival of lens precursor cells (Zhao *et al.* 2008) and promotes lens fiber cell differentiation *in vivo* (Madakashira *et al.* 2012). Studies of Wnt (Smith *et al.* 2005), BMP (Faber *et al.* 2001; Rajagopal *et al.* 2008, 009), Notch (Jia *et al.* 2007; Rowan *et al.* 2008; Le *et al.* 2009; Saravanamuthu *et al.* 2009, 2012), and TGF-β (Saika *et al.* 2001; Beebe *et al.* 2004) signaling in mouse demonstrated a number of specific roles of these signaling pathways in lens fiber cell differentiation. Recent studies using chick lens epithelial cells generated data suggesting a specific cross-talk between FGF and BMP signaling (Boswell *et al.* 2008a,b) and its requirement for cell-cycle exit of lens cells *in vivo* (Jarrin *et al.* 2012). Finally, human embryonic stem cells can be differentiated into lens progenitor-like cells by the use of a combination of BMP4, BMP7, and FGF2 (Yang *et al.* 2010). In this system, FGF2 was both essential and sufficient for the formation of more differentiated structures, the lentoid bodies (Yang *et al.* 2010).

Nevertheless, given the complexity of these pathways, additional studies on the lens fiber differentiation are needed to understand hierarchy and contribution of these molecular networks to the lens fiber cell differentiation (Smith *et al.* 2010). Lens-specific inactivation of Dicer1 in the prospective lens placode demonstrated that miRNAs plays multiple functions during lens formation (Li and Piatigorsky 2009). In a genome-wide study, authors identified the expression of at least 20 miRNAs in mouse lens (Karali *et al.* 2010); however, additional miRNAs expressed in the lens remain to be discovered. In terms of individual miRNAs, it has been shown recently that miR-204 controls multiple aspects of lens formation and differentiation and its expression is Pax6-dependent (Conte *et al.* 2010; Avellino *et al.* 2013; Shaham *et al.* 2013). Two specific miRNAs (miR-7a and miR-9) regulate expression of Pax6 during mouse neurogenesis (Shibata *et al.* 2011; de Chevigny *et al.* 2012; Zhao *et al.* 2012). Although Pax6 has been established as a key regulator of lens differentiation (Cvekl and Piatigorsky 1996; Shaham *et al.* 2012), roles of these and other miRNAs in the lens are at present unknown (Conte *et al.* 2013).

Differentiation of cultured rat lens explants has been used as a powerful system to study mammalian lens fiber cell differentiation for over two decades (McAvoy and Chamberlain 1989; Zelenka *et al.* 2009; West-Mays *et al.* 2010). Different concentrations of FGF2 induce proliferation, migration, and terminal differentiation of lens explants. At 50−100 ng/mL of FGF2, this system recapitulates major features of lens fiber cell differentiation, including cell elongation, expression, and accumulation of crystallins in approximate synchrony during a period of days to weeks, and gradual degradation of cytoplasmic organelles including the nuclei (McAvoy and Chamberlain 1989; Zelenka *et al.* 2009). Herein, role of miRNAs during lens fiber cell differentiation was assessed *in vivo* through conditional inactivation of Dicer1 using lens-specific Cre driver. The range and spectrum of FGF2-dependent responses in the differentiating lens explant system were evaluated through integrated mRNA and miRNA expression profiling. The results of present studies are applicable not only for lens fiber cell differentiation but also for understanding how FGF signaling could regulate cellular differentiation in other systems.

## Materials and Methods

### Conditional inactivation of Dicer1 in the lens

Dicer1^flox/flox^ mice (Harfe *et al.* 2005) were mated with MLR10-Cre transgenic mice (Zhao *et al.* 2004), and the progeny were crossed to generate litters containing homozygous floxed alleles and heterozygous for Cre transgene. Mice genotyping was performed as we described previously (Davis *et al.* 2011). At noon, the vaginal plug was observed and was considered as E0.5 of embryogenesis. Animal husbandry and experimental procedures were conducted in accordance with the approved protocol of the Sackler School of Medicine Animal Institute Committee and the ARVO Statement for the use of animals in Ophthalmic and Vision Research.

### Antibodies and histological analysis

Animals were killed by CO_2_, and either the embryos were dissected from pregnant females or whole eye balls were removed from postnatal animals. Tissues were then fixed in 10% neutral buffered paraformaldehyde overnight at 4°, processed, and embedded in paraffin. Paraffin sections (10-μm) were stained with hematoxylin and eosin using standard procedures. Immunofluorescence analysis was performed on paraffin sections as previously described (Ashery-Padan *et al.* 2000) using the following primary antibodies: rabbit anti-Crystallin γ (1:50, sc-22764; Santa Cruz Biotechnology, Inc.), rabbit anticleaved caspase 3 (1:300, #9661; Cell Signaling Technology) and mouse anti-E-cadherin (1:250, #6101982; BD). Secondary antibodies were conjugated to Alexa488/594 donkey anti-mouse/rabbit (1:1000, A-21202/ A-21207; Invitrogen) or Alexa488 donkey anti-mouse/goat (1:1000, A-21202/A-11055; Invitrogen). Nuclei were visualized with 4′,6-diamidine-2-phenylidole-dihydrochloride (DAPI, 0.1 μg/ml; Sigma-Aldrich, St. Louis, MO).

### Rat lens explants, oligonucleotide microarrays, and mRNA expression profiling

Primary rat lens explants were prepared using 3-d-old rat lenses as described elsewhere (Zelenka *et al.* 2009). Six explants were seeded per dish, grown overnight in the presence of 5 ng/mL FGF2 to promote their proliferation and survival, and induced to differentiate by a concentration of 100 ng/mL FGF2 (Sigma-Aldrich). After the treatments, the lens explants were stored in RNA Later (Ambion, Woodlands, TX). RNA isolations were performed using the RNeasy Mini and miRNeasy Kits and RNase-Free DNase set and (QIAGEN, Valencia, CA). RNA quality was assessed using the Agilent 2100 Bioanalyzer with the Nano LabChip Kit (Agilent Technologies; Palo Alto, CA) following the manufacturer’s instructions. Two sets of RNAs from different cultures were prepared for the microarray analyses. cDNA synthesis and amplifications were performed with Ovation RNA Amplification System V2 (Nugen, San Carlos, CA) using 50 ng of total RNA per sample. Amplified cDNAs were cleaned and purified with DNA clean and Concentrator -25 kit (Zymo Research, Orange, CA). Fragmentation and labeling was performed using the FL Ovation cDNA Biotin Module V2 (Nugen) according to manufacturer’s instructions. The two sets of samples were subsequently hybridized on Rat Genome 430A 2.0 Arrays (Affymetrix, Santa Clara, CA) following the manufacturer’s specification.

### miRNA profiling

Aliquots of identical total RNA biological duplicate preparations used for Affymetrix GeneChip profiling were used for miRNA profiling using the TaqMan Low-Density Array System (TLDA; Life Technologies). We converted the miRNA contents in 50 ng of total RNA to cDNA using the Megaplex RT pool system, preamplified it using the Megaplex Preamp Primers, and analyzed it on TaqMan Array Rodent MicroRNA cards A and B, version 2, using the ABI 7900HT SDS qPCR system. The resulting profiles were normalized to the *C*_t_ values of the internal mammalian U6 probe. The normalized profiles were imported to GeneSpring GX11 (Agilent, Santa Clara, CA) for further analysis.

### Bioinformatic tools and statistical filtering of mRNA microarray and miRNA TLDA results

Time−course analysis of FGF2 response was performed in biological duplicate sets (n = 2) of each time point. Robust multichip average−normalized intensities (http://rmaexpress.bmbolstadcom) were extracted from Affymetrix CEL files. Analysis of variance (*P* < 0.05) and Pavlidis Template Matching using the TIGR Multiexperiment Viewer of the TM4 (Saeed *et al.* 2003) were used to identify reproducibly modulated transcripts and patterns of inverse correlation with the time-matched miRNA profiles. The miRNA data were analyzed by self-organizing maps, a semisupervised analysis tool available from the TM4 TIGR Multiexperiment Viewer to determine major miRNA expression profile groups. Primary data from this study were deposited in the NCBI Gene Expression Omnibus database under accession number GSE50604. The Gene ontology (GO) and KEGG pathway functional annotations were performed using the NIH web-based tool DAVID (the Database for Annotation, Visualization and Integrated Discovery; Huang da *et al.* 2009).

### Identification of miRNA targets

The targets of modulated miRNAs in the modulated mRNA set were identified by a combination of an R script interphasing with TargetScan 5.0 to import predicted targets and by integrated prediction databases in miRWalk (Dweep *et al.* 2011). The inverse miRNA:mRNA target correlation patterns were determined using the miRNA analysis tool in the Ingenuity Pathway Analysis software (Ingenuity Systems, Redwood City, CA). Overall connectivity of each miRNA with its gene targets and of each target gene with upstream miRNA regulators was calculated and used for ranking of the top miRNA-dependent genes that were then visualized as reduced complexity networks using the Cytoscape software (Cline *et al.* 2007; Smoot *et al.* 2011).

### Transient transfections and luciferase reporter assays

TargetScan was used to identify binding sites in approximately the 3-kb mouse c-Maf 3′-UTR region. The wild-type and mutated DNA fragments were synthesized by Genescript (Piscataway, NJ) and subcloned into the pMIR-REPORT luciferase vector (part number: AM5795; Applied Biosystems). Mouse αTN4-1 lens epithelial cells were cultured as described elsewhere (Yang and Cvekl 2005). The transfection were performed using Lipofectamine 2000 (Invitrogen) with 150 ng of 3′-UTR reporter construct, 20 ng of *Renilla* luciferase normalization vector, and 10 pmol of miRNA (miR) mimics (QIAGEN). The miRNA mimics are double-stranded RNAs that resemble mature miRNAs after being transfected into cells. After the transfection, cells were grown for 24−30 hr. The cell lysis and the reporter activities were conducted as described by Promega’s Dual-Luciferase Reporter Assay System. Each transfection was conducted as triplicate and repeated twice.

### miRNA *in situ* hybridization (ISH)

miRNA ISH was performed as described previously (Karali *et al.* 2010). For detection of the mature miRNA sequence, 5′DIG prelabeled miRCURY LNA miRNA Detection Probes (Exiqon, Vedbaek, Denmark) were used at a final concentration of 50 nM. Probes were hybridized at 42°. For miRNA ISH, hsa-miR-20b miRCURY LNA detection probe (working concentration 7 nM, Exiqon) was hybridized to frozen sections as described previously (Shaham *et al.* 2013).

## Results

### Targeted deletion of Dicer1 causes multiple lens developmental abnormalities

**Figure 1:**
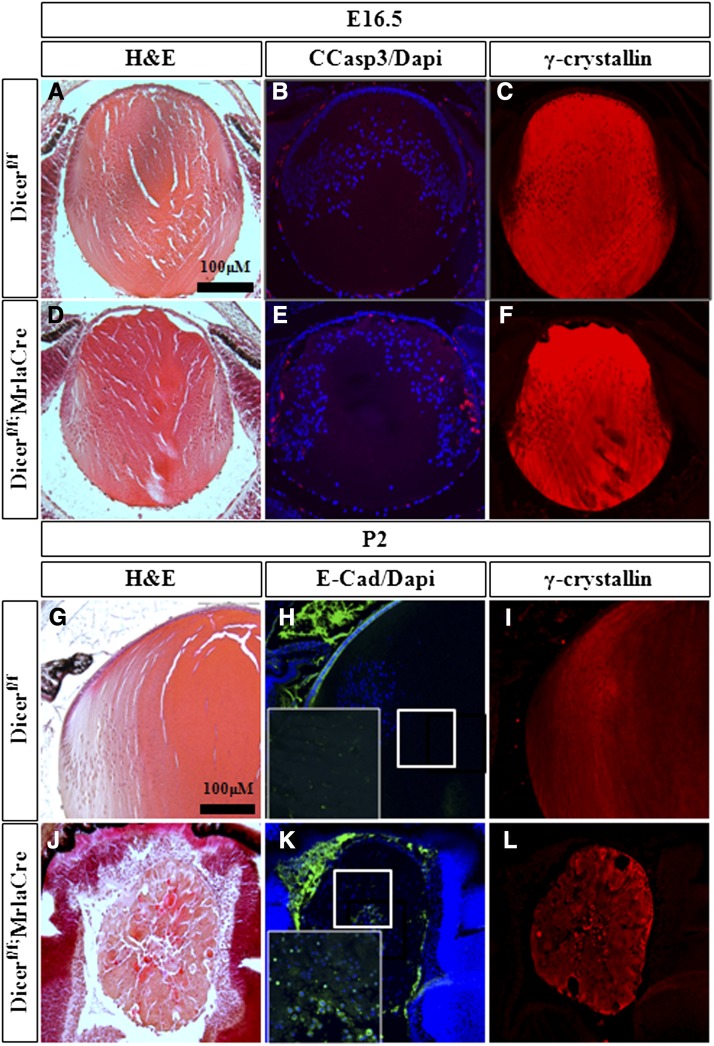
Lens fiber cell differentiation is disrupted upon conditional inactivation of Dicer1. Analysis of lens development at E16.5 (A−F) and P2 (G−L). in control *Dicer1^flox/flox^* (A−C, G−I) and *Dicer1^flox/flox^*; *MLR10-Cre* mutant (D−F, J−L). The analysis presented includes hematoxylin and eosin staining (A, D, G, J), immunofluorescence staining for cleaved caspase 3 (B, E), E-cad (H, K), and γ-crystallins (C, F, I, L). Scale bar = 100 μm.

Previous conditional inactivation of Dicer1 at the lens placode stage (*Dicer1^flox/flox^;LeCre*) supports the requirements for miRNAs during lens development (Li and Piatigorsky 2009). However, in *Dicer1^flox/flox^;LeCre* mutants, the lens epithelium was diminished at embryonic day 14.5 (E14.5), and lens tissue was lost by postnatal day 0 (P0), thus providing limited insight on the potential role of miRNAs during lens fiber differentiation. To substantiate the role of miRNAs during secondary fiber differentiation, we inactivated *Dicer1^flox^* by using the *MLR10-Cre* line, which is active after primary fibers formed and before the onset of secondary fiber differentiation (Zhao *et al.* 2004; Harfe *et al.* 2005; Shaham *et al.* 2009). Consistent with the later onset of MRL10-Cre and the stability of miRNAs, the phenotype of *Dicer1^flox/flox^;MLR10-Cre* lens was evident from around E16.5 with elevation of apoptosis in mutant lens based on the presence of cleaved caspase 3, which is not detected in the control littermates (Figure 1, B and E; *Dicer1^flox/flox^*). We did not detect any significant change in expression of γ-, αA-, αB-, and β-crystallins (Figure 1, C and F and data not shown). Thus, miRNAs appear to be essential for survival of lens cells, although their role in gross regulation of crystallin genes is probably not substantial at E16.5.

The lens phenotype of the *Dicer1^flox/flox^;MLR10-Cre* was prominent during later stages of development. At postnatal day 2 (P2), the *Dicer1^flox/flox^;MLR10-Cre* lens fiber morphology was disrupted (Figure 1, G and J), and lens epithelium was diminished on the basis of reduced expression of E-cadherin (Figure 1, H and K). In addition, the *Dicer1^flox/flox^;MLR10-Cre* lens fiber cells, although expressed several crystallins (not shown), failed to properly elongate. In contrast to the organelle-free zone that is apparent in the control lenses (inset of DAPI staining; Figure 1, H and K), the nuclei in Dicer1-depleted lenses were retained. These results demonstrate that Dicer1, and, inferring from it, miRNAs, are required for survival of the lens epithelium and for terminal differentiation of lens fiber cells. These roles for miRNAs raise the possibility that there is a functional link between FGF signaling as a major pathway that controls lens fiber cell differentiation, survival of lens cells, and miRNA-dependent lens morphogenesis. To address this link experimentally, we used an *in vitro* differentiation system that allows systemic analysis of these processes.

### RNA/transcriptome and miRNA/miRNome expression profiling: a global analysis

**Figure 2:**
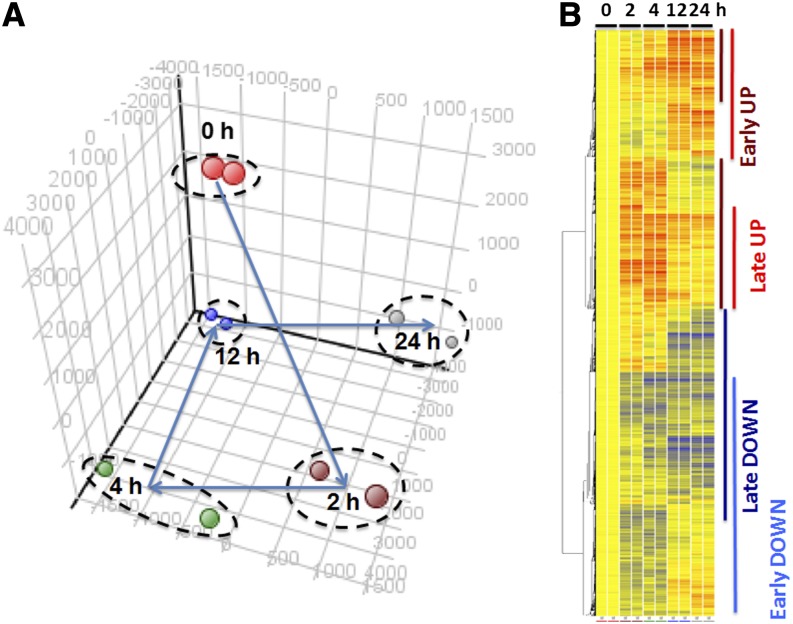
Global analysis of RNA expression data during FGF2-induced lens fiber cell differentiation. (A) PCA of the RNA expression data. (B) Clustering of RNA expression profiles visualized as heat map diagram. Vertical bars: “Early UP” group, dark red; “Late UP” group, red; “Early DOWN” group, light blue; “Late DOWN” group, dark blue. The analysis of miRNAs is shown in Figure S1.

Previous studies in which investigators used the rat lens explant system showed that early features of lens fiber cell differentiation, such as exit from the cell cycle and expression of α-crystallins, are detectable within the interval of 12−24 hr after the treatment of cells with 100 ng/mL FGF2 (Leenders *et al.* 1997; Golestaneh *et al.* 2004; O’Connor *et al.* 2008). Thus, we selected 2, 4, 12, and 24 hr as individual time points after the treatment for the global RNA expression analysis to identify changes in transcriptome and miRNome profiles related to the “early” response phase, such as regulation of the cell cycle exit (2 and 4 hr), followed by “later” response phase, including the onset of lens cell differentiation (12- and 24-hr time points), respectively. As described in the section *Materials and Methods*, the explants were first grown overnight in the presence of a low concentration of 5 ng/mL of FGF2 to support their proliferation and survival (Zelenka *et al.* 2009). Two sets of biological replicates were used for DNA microarray hybridization with the Affymetrix Rat Genome 430A 2.0 Arrays. The statistical and bioinformatics analyses were performed as described in the section *Materials and Methods*. Initially, we identified 5544, 4827, 4432, and 5347 differentially expressed transcripts between the differentiating and control lens cells at 2-, 4-, 12-, and 24-hr time points from a total number of over 22,000 rat genes represented on the array, respectively. Notably, the number of genes decreased by approximately 40% in each category if a fold-change cut off (at least 1.25-fold up- or down-regulation) was applied (see Table 1). Principal component analysis (PCA) of the data revealed a dramatic shift between time points 0 and 2 hr followed by a stairway-like climb into a novel transcriptome state of cells treated for 12 and 24 hr (see Figure 2A). Unsupervised hierarchical clustering of those 11,439 individual probe sets/transcripts identified a series of blocks of coregulated transcripts (see Figure 2B). The major blocks included groups of transcripts up-regulated at 2 and 4 hr that either stayed up-regulated or were down-regulated. Conversely, other blocks contained transcripts down-regulated at the “early” time points, followed by their continuous down-regulation or restoration, *i.e.*, return to their original expression levels. Both the PCA and hierarchical clustering identified notable similarities between transcript levels at the either “early” or “late” response phases after the treatment with FGF2. Therefore, we concluded that gene expression profiles between “early” and “late” response phases are markedly different and likely represent distinct cellular processes elicited by FGF2.

**Table 1:** Global analysis of RNAs, miRNAs, and miRNA:mRNA pairs

mRNA Profile	mRNA PTM/FC > 1.25, n	miRNAs	miRNAs, n	miRNA-Regulated mRNAs (FC > 1.25)	Inversely Regulated miRNA:mRNA sets*^a^*)
Early down	3051	Early up	50	665 (22% down)	Early miRNA:mRNAs
Early up	3321	Early down	16	376 (11% up)	1041
Late down	3028	Late up	36	458 (15% down)	Late miRNA:mRNA
Late up	2839	Late down	29	380 (13% up)	838

miRNA, microRNA.

a Overlap Early miRNA:mRNA and Late miRNA:mRNA = 99.

miRNA profiling was evaluated simultaneously using the rodent TLDA v2 ABI system at the same time points. The initial analysis revealed a total number of 204 up- and down-regulated miRNAs. Individual expression profiles of these miRNAs were preanalyzed by self-organizing maps followed by template matching on duplicate temporal profiles to identify the main miRNAs expression profiles (see Supporting Information, Figure S1). Like for the transcript analysis, both up- and down-regulated miRNAs clustered into blocks of coregulated miRNAs. For follow-up studies, we focused on those miRNAs that were modulated in at least two time points and this selection led to a list of 131 miRNAs that were subsequently analyzed (see File S1). These 50, 16, 36, and 29 individual miRNAs were classified as “early-up,” “early-down,” “late-up,” and “late-down,” respectively (Table 1). Table 2 shows a complete list of these 131 miRNAs and their temporal classification. Analysis of their fold-changes revealed that 38 (29%), 45 (34%), and 47 (35%) of these miRNAs were induced at least 3-fold, between 2- to 3-fold, and reduced by at least a factor of 2, compared with the zero time point, respectively. Taken together, the miRNome and transcriptome data suggest that the FGF2-mediated modulation of the miRNome may have a significant impact on the posttranscriptional regulation of the lens transcriptome that governs lens differentiation.

**Table 2:** A complete list 131 FGF2-regulated miRNAs and their expression profile classification

Class	Member miRNA
Early UP (n = 50)	let-7f, let-7i*, miR-100, miR-129-3p, miR-133b, miR-134, miR-137, miR-154*, miR-17-3p, miR-186*, miR-191*, miR-193*, miR-200c, miR-205, miR-21*, miR-219, miR-219-1-3p, miR-23a*, miR-27a*, miR-27b*, miR-29a*, miR-29b*, miR-31, miR-31*, miR-323-3p, miR-328, miR-333, miR-336, miR-342-5p, miR-362-3p, miR-376a, miR-376b*, miR-377, miR-380-5p, miR-381, miR-382, miR-383, miR-409-3p, miR-411*, miR-421, miR-434-5p, miR-455, miR-463, miR-485*, miR-495, miR-543, miR-667, miR-704, miR-879*, miR-9
Early DOWN (n = 16)	miR-125b-3p, miR-135a*, miR-141, miR-193, miR-296-3p, miR-375, miR-449a, miR-494, miR-503, miR-675-3p, miR-682, miR-685, miR-694, miR-720, miR-801, miR-804
Late UP (n = 36)	miR-106a, miR-10a, miR-132, miR-135b, miR-138*, miR-152, miR-155, miR-181a, miR-182, miR-184, miR-191, miR-203, miR-21, miR-210, miR-222, miR-290-5p, miR-296-5p, miR-298, miR-29a, miR-29b, miR-29c, miR-324-3p, miR-324-5p, miR-329, miR-345-3p, miR-34a, miR-34b-3p, miR-369-3p, miR-376b, miR-376c, miR-379, miR-449c, miR-708, miR-345-3p, miR-743a, miR-758
Late DOWN (n = 29)	let-7g*, miR-106b*, miR-125a-3p, miR-125b-5p, miR-126-3p, miR-142-3p, miR-142-5p, miR-143, miR-145, miR-197, miR-20b, miR-214, miR-214*, miR-217, miR-297c, miR-301a, miR-339-5p, miR-342-3p, miR-496, miR-539, miR-542-3p, miR-542-5p, miR-672, miR-690, miR-7a, miR-7b, miR-339-3p, miR-351, miR-450a

Note: The star (*) labeled miRNA* represents the complementary (“passenger”) strand formed during the duplex cleavage that produces the 21-nt long mature miRNA (the guide strand). FGF2, fibroblast growth factor 2; miRNA, microRNA.

To test this hypothesis, candidate miRNA targets were identified *in silico* through a combination of TargetScan 5.0 and integrated prediction databases in miRWalk and by inverse miRNA:mRNA correlation patterns as described in the section *Materials and Methods*. Initially, we performed analysis in four groups corresponding to the individual time points, 2, 4, 12, and 24 hr. We found that the “early” and “late” miRNA:mRNA pairing revealed almost identical sets of miRNAs. Thus, for simplification of the subsequent analyses, we formed two groups: “early” and “late.” In the “early” group, 50 up-regulated miRNAs were linked to 665 target mRNAs down-regulated in the system as shown in Table 1. Similarly, 16 down-regulated miRNAs were connected with 376 up-regulated target mRNAs. In “late” group, 36 up- and 29 down-regulated miRNAs were linked with 458 and 380 inversely correlated mRNAs, respectively. Collectively, we identified a total number of 1041 and 838 mRNAs that are possibly regulated by FGF2-dependent miRNAs (Table 1) in the “early” and “late” response phases during FGF2-induced lens differentiation process, respectively.

**Figure 3:**
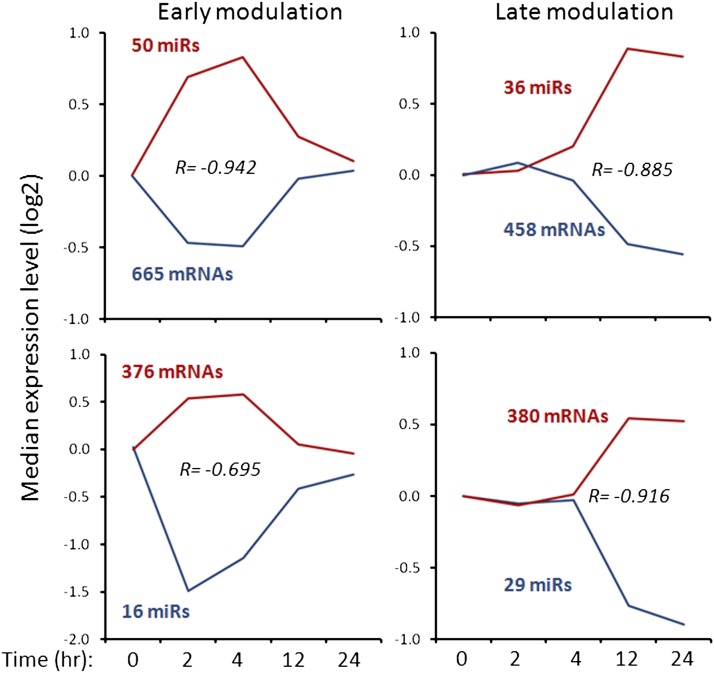
Correlation between RNA and miRNA expression profiles during FGF2-induced lens fiber cell differentiation. Global analysis (Table 1) identified specific up- and down-regulated miRNAs at individual time points followed by computational programs to identify their target genes and analysis of expression profiles of these presumptive RNA targets. Median log2 values for each expression profile group are plotted as representative patterns with the underlying numbers of miRNAs and mRNAs indicated. R = Pearson product moment correlation coefficient indicates inverse correlation between the median-based profiles for miRNAs and mRNAs. The inversely correlated genes are shown in File S1, A and B.

The miRNA:mRNA correlations were further quantitatively evaluated by calculating the Pearson product moment correlation coefficient (R) for all four miRNA:mRNA sets. The R values (see Figure 3) range from −0.695 to −0.942, indicating that the experimental system contains inversely correlated miRNA:mRNA pairs. In addition, these graphs confirmed that up-regulated miRNAs at both “early” time points returned to their original expression levels between 12 and 24 hr (Figure 2, early modulation). Similarly, “late” modulated miRNAs influence transcript levels at both 12 and 24 hr after the initial treatment. Collectively, the *in silico* analysis suggested four sets of miRNA-regulated mRNA, including a total number of 1879 transcripts. Among them, a minor fraction of 99 (5.3%) are transcripts changed at both “early” and “late” stages. Taken together, these analyses further confirm the data shown in Figure 2 indicating that the processes induced by FGF2 are substantially different at the “early” and “late” phases of the differentiation.

### Initial functional and subcellular analysis of miRNA-regulated mRNAs

**Figure 4:**
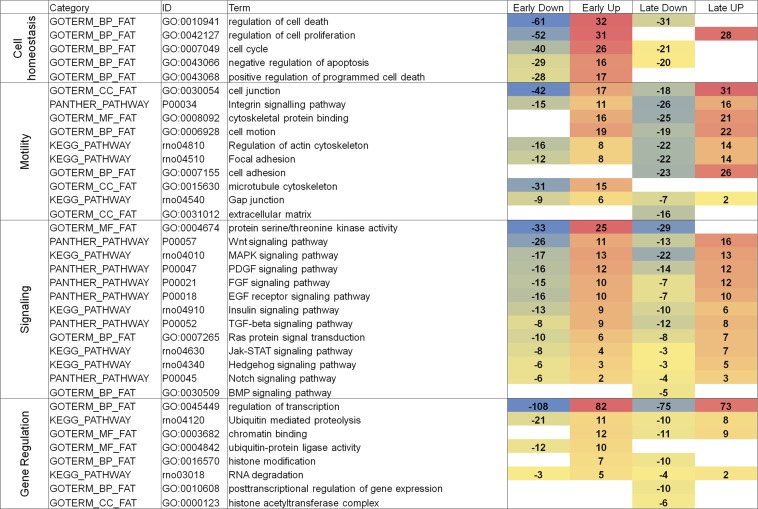
A summary of major GO categories of miRNA targets. Four individual groups of RNA targets (665, 376, 458, and 380; see Table 1) were analyzed by the use of DAVID GO databases and four groups, including Cell homeostasis, Motility, Signaling, and Gene Regulation, related to lens fiber cell biology are shown. Relative levels of down- and up-regulated genes are shown with negative and no sign, respectively. A total number of genes from each of four columns was calculated and used to rank the “GO term” from the greatest to lowest numbers of regulated genes. The individual genes are shown in File S2.

The individual genes from the 1041 and 838 gene lists (Table 1) were imported into the GO (Biological Process, Molecular Function, and Cellular Compartment) and KEGG Pathway functional annotations to link the FGF2-regulated/miRNA-dependent genes with the function and subcellular localization. The miRNA-dependent mRNAs distributed primarily among the groups of nuclear proteins, cell-surface receptors, and intracellular signaling proteins (data not shown), suggesting a role for FGF-induced miRNAs as main effectors of rapid remodeling of gene expression programs in response to external stimulus. The GO and KEGG Pathway functional classification identified four “high” GO levels, including Cell Homeostasis, Motility, “Signaling,” and Gene Regulation, and their numerous subcategories (see Figure 4). They include “Regulation of cell death,” “Regulation of cell proliferation,” and “Regulation of cell cycle”; all these categories were shown to be regulated by FGF2 in rat lens cell explants using cell biology studies (Lovicu and McAvoy 2005; Griep 2006; West-Mays *et al.* 2010). The “Cell homeostasis” group contains more than a 3-times greater number of genes in the “early” compared with the “late” response phase. This category includes “Regulation of cell death” and “Regulation of cell proliferation.” These data suggest that FGF2 controls the cell-cycle machinery/survival pathways as soon as the level of FGF2 is increased to induce cellular differentiation (see section below: *FGF2-induced miRNAs are key major regulators of cell-cycle arrest*). In contrast, the “Motility group” is represented in both “early” and “late” stages, the number of regulated genes is ~1.4 greater in the “late” group.

The most notable finding is a global impact of activated FGF signaling toward other signaling pathways, including Wnt, MAPK, platelet-derived growth factor, epidermal growth factor, insulin, TGF-β, Ras protein, Janus kinase and signal transducer and activator of transcription, hedgehog, and Notch, mediated by miRNAs at both “early” and “late” response phases of the lens differentiation cultures. In contrast, only a few genes within the BMP signaling pathway appear be regulated via FGF2-modulated miRNAs (Figure 4). In the category “Gene Regulation,” represented by eight functional categories, we found two common denominators. Four groups, including “Ubiquitin mediated proteolysis,” “Ubiquitin-protein ligase activity,” “RNA degradation,” and “Posttransriptional regulation of gene expression,” are linked together through the degradation of proteins and RNAs used to accommodate the switch between the “early” and “late” stages of the FGF2-induced processes. Three groups, including “Chromatin binding,” “Histone modification,” and “Histone acetyltransferase complex,” relate to gene regulation at the level of chromatin.

### miRNA:mRNA connectivity, miRNA ranking, and initial network analysis

**Figure 5:**
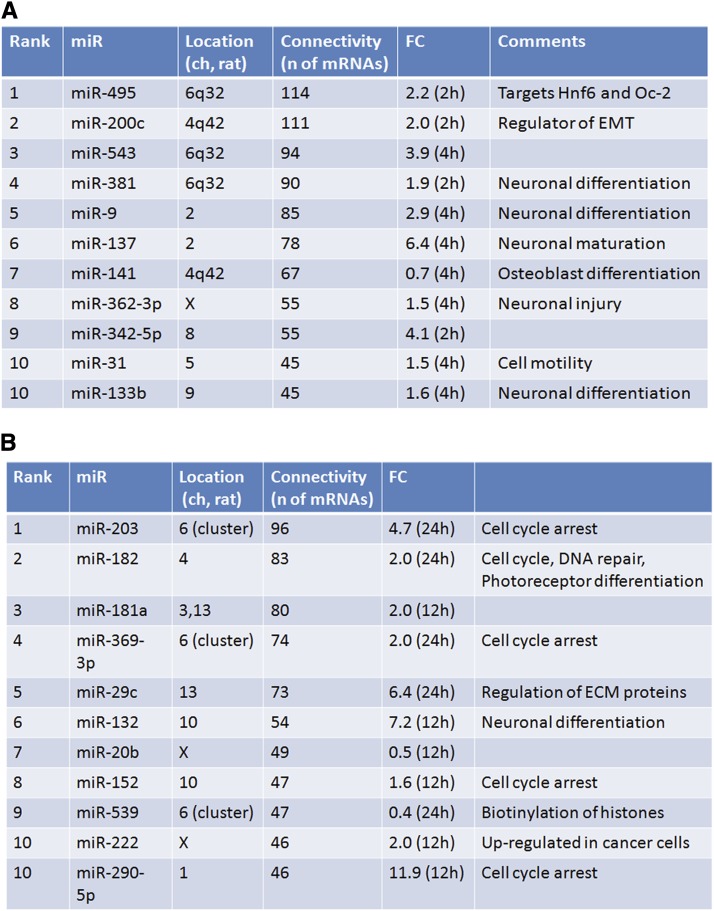
A summary analysis of miRNA connectivity and their FGF2-mediated inducibility. (A) Top 10 ranking of FGF2-regulated “early” miRNAs depending on the number of their predicted/regulated target genes. (B) Top 10 ranking of FGF2-regulated “late” miRNAs depending on the number of their predicted/regulated target genes. The complete lists of genes/miRNAs are shown in File S3, A and B.

It has been shown earlier that a single miRNA can recognize hundreds of target mRNAs and that multiple miRNAs can regulate expression of genes with joint roles in a specific pathway and connect multiple genes into highly complex regulatory hubs (Ivey and Srivastava 2010; Pauli *et al.* 2011). To address miRNA connectivity in the present study, we used human TargetScan in combination with ingenuity pathway analysis because this platform allows identification of inversely correlated miRNA and mRNA patterns in a batch mode with adjustable prediction score level. Using the starting numbers of 66 “early” and 65 “late” miRNAs, we found that this procedure mapped 31 and 45 miRNAs, with 549 and 531 target mRNAs, respectively. In sum, these 76 miRNAs regulated 1080 mRNAs. The top-ten most connected miRNAs, divided in “early” and “late” sets, are shown in Figure 5, A and B, respectively. The total number of their target genes is between 114 (1st rank, “early” miR-495) and 45 (10th rank, “early” miR-31 and miR-133b) connections. Notably, a number of genes appeared to be regulated by more than ten distinct miRNAs. For example, Cpsf6 and Tnrc6b (“early” group) are regulated by 11 distinct miRNAs. In the “late” group, Aak1, Cnot6, and Nfat5 were regulated by 16, 11, and 13 different FGF2-dependent miRNAs, respectively. From this analysis, we concluded that the present experimental set is enriched for specific mRNAs that are coregulated by a large number of distinct FGF2-regulated miRNAs, raising the possibility that these novel FGF2/miRNA-dependent gene regulatory networks (GRNs) play major roles during lens fiber cell differentiation.

**Figure 6:**
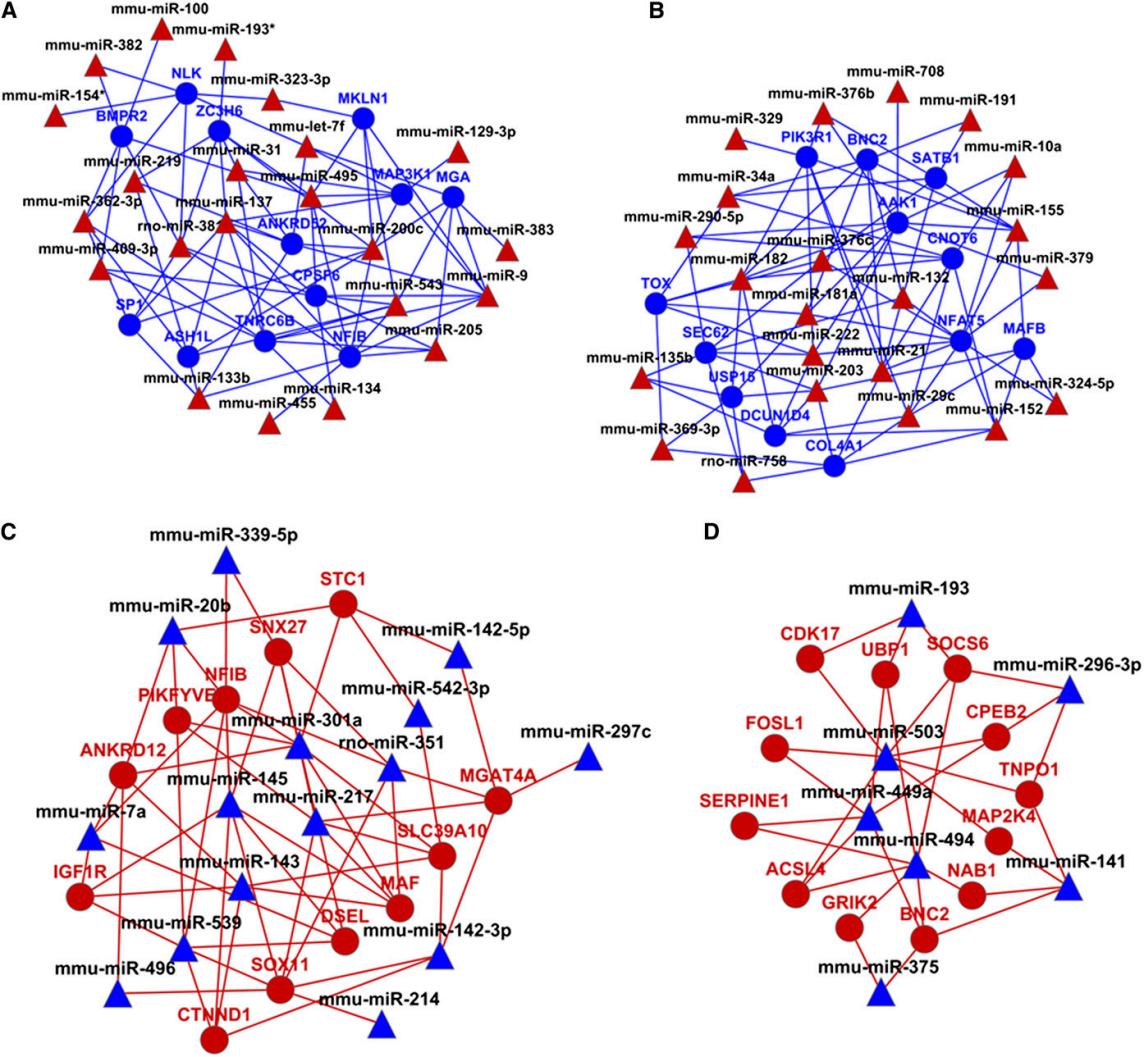
Four sets of global GRNs. (A) “Early” up-miRNA:down-regulated mRNA. (B) “Late” up-miRNA:down-regulated mRNA. (C) “Early” down-miRNA:up-regulated mRNA. (D) “Late” down-miRNA:up-regulated mRNA. The diagrams were created using Cytoscape program as described in the *Materials and Methods* using only high-scoring and experimentally observed predictions. miRNA, triangles; mRNAs, circles; up-regulated (red); down-regulated (blue).

We next evaluated the global GRNs by using the Cytoscape software (Cline *et al.* 2007; Smoot *et al.* 2011). We found that novel biological information can be “clearly” displayed if 12 top-ranking, most-connected genes with high target prediction score or an experimentally validated relationship with the upstream miRNAs, are used as an input. Following this approach, four connectivity diagrams were obtained for “early” up-miRNAs:down-regulated mRNA, “late” up-miRNAs:down-regulated mRNA, “early” down-miRNAs:up-regulated mRNA, and “late” down-miRNAs:up-regulated mRNA, as shown in Figure 6, A−D, respectively. The diagrams for “early” repressed and activated GRNs generated three distinct patterns of coregulated mRNAs. In Figure 6A, the 12 target repressed mRNA targets formed three clusters that were regulated by 22 distinct miRNAs. In Figure 6, B and C, the 12 mRNAs were coregulated by 15 and 22 miRNAs and were arranged as two distinct groups. In Figure 6D, the 12 up-regulated mRNAs were under the control of seven down-regulated miRNAs.

The most connected miRNA identified here through the 12 top-ranking transcripts, including miR-495, miR-200c, miR-543, miR-381, and miR-9 (Figure 6A), retained their high-connectivity positions as identified by independent analysis shown earlier in Figure 6A. Similarly, the highest ranking “late”-induced miRNAs, including miR-203, miR-182, miR-181a, miR-369-3p, and miR-29c (Figure 6B), also make the greatest number of connections with the 12 genes analyzed in Figure 6B. We conclude that the individual miRNA:mRNA pairs are components of larger networks formed through joint coregulation of specific mRNA by multiple FGF2-regulated miRNAs during lens explant differentiation. In addition, the present data also suggest that there are small groups of individual genes targeted by three or more coregulated miRNAs.

### Individual GRNs

To get additional insights into the “complex” GRNs (see Figure 6), and to demonstrate significance of an “increased” miRNA:mRNA connectivity, *i.e.*, defined here as three or more miRNAs regulating at least two functionally related genes, we reasoned that genes regulated by miRNAs in *both* “early” and “late” groups represent the major component of FGF2/miRNA-dependent regulation of the lens fiber cell differentiation. To test this hypothesis, we identified five genes, including Nfib, Nfat5/OREBP, c-Maf, Ets1, and N-Myc, as the genes regulated by the greatest total number of 18 (10+8), 14 (1+13), 13 (8+5), 8 (1+7), and 8 (5+3) differentiation stage−specific miRNAs, respectively. Within this group, three genes encode DNA-binding transcription factors with established functions in lens differentiation (c-Maf, Nfat5/OREBP, and N-Myc; Harris *et al.* 1992; Morgenbesser *et al.* 1995; Wang *et al.* 2005; Yang *et al.* 2006; Martins *et al.* 2008; Xie and Cvekl 2011). Interestingly, Ets1 is expressed throughout the mouse embryonic E14 lens (Kola *et al.* 1993), and it has been shown to mediate FGF/MAPK signaling in pituitary cells (Schweppe *et al.* 1997). Loss of Nfib in lung mesenchyme and epithelium perturbs FGF signaling (Hsu *et al.* 2011). In addition, Nfib also functions in the Pax6-dependent GRN in forebrain development (Mason *et al.* 2009). Pax6 has been shown as the key regulatory gene of lens development (Cvekl and Piatigorsky 1996; Shaham *et al.* 2012). Taken together, Nfib, Nfat5, c-Maf, Ets1, and N-Myc represent a subgroup of highly miRNA-connected regulatory genes with either proven or potential function(s) in lens differentiation.

We next divided these transcription factors into an “early” (c-Maf, N-Myc, and Nfib) and a “late” (c-Maf, Ets1, N-Myc, Nfat5/OREBP, and Nfib) groups. Interestingly, in the “early” and “late” response phases, Ets1 and Nfat5, are targeted by only a single miRNA, *i.e.*, miR-193 and miR-494, respectively. We next analyzed “early” and “late” connectivities. We found that seven miRNAs, including miR-9, miR-137, miR-200c, miR-381, miR-455, miR-495, and miR-543, target at least two “early” genes examined (*i.e.*, c-Maf, N-Myc, and Nfib). Notably, miR-381, miR-495, and miR-543 form a miRNA-gene cluster on rat chromosome 6, and its syntenic regions on mouse and human chromosome 12 and 14, respectively (Sewer *et al.* 2005). In human, the DLK1-DIO3 genomic imprinted miRNA cluster at 14q32.2 defines an important region related to stem cell biology and cancer (Agueli *et al.* 2010; Luk *et al.* 2011).

**Figure 7:**
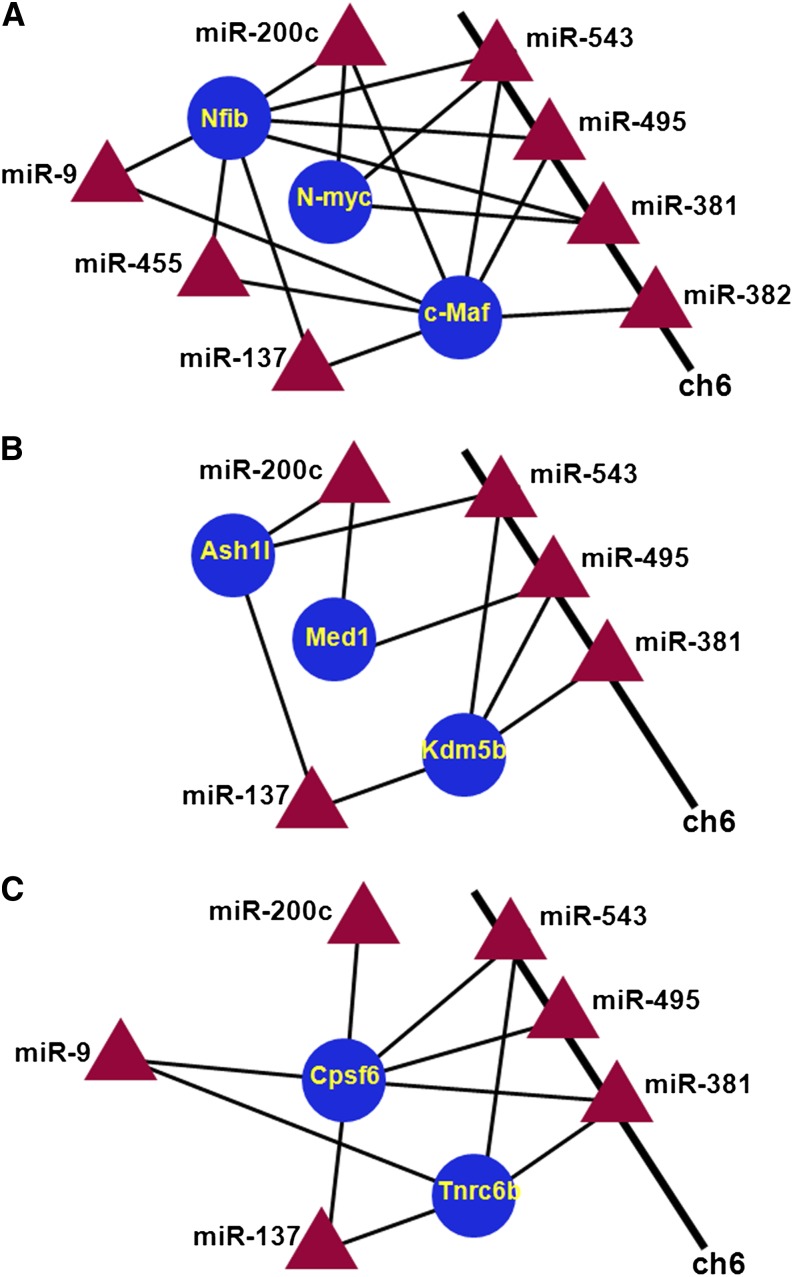
An “early” connectivity network. Seven miRNAs, including miR-9, miR-137, miR-200c, miR-381, miR-455, miR-495, and miR-543, and connections to specific functional groups of genes are shown. (A) Connections to the group of three most-connected and regulated transcription factors (c-Maf, Nfib, and N-Myc). (B) Chromatin regulation (Ash1l, Med1/PBP, and 20 Kdm5b/Jarid1b/Plu1). (C) RNA metabolism (Cpsf6 and Tnrc6b). The miR-381, miR-495, miR-543, and miR-382 form a miRNA-gene cluster on rat chromosome 6q32. miRNA, triangles; mRNAs, circles; up-regulated (red); down-regulated (blue).

We next created a diagram of c-Maf, N-Myc, and Nfib (Figure 7A). This connectivity diagram showed the multitude of regulatory interactions between these seven miRNAs and their three target mRNAs because miR-382 is also FGF2-regulated, targets c-Maf, resides at the rat chromosome 6 gene cluster of 61 miRNAs, and was included in Figure 7A. Next, we found additional 22 genes that follow this commonly used regulatory mechanism of these seven miRNAs. Functional classification via the use of published studies led to the formation of seven smaller functional groups containing between two and six genes: RNA metabolism (Cpsf6 and Tnrc6b), E3 ligases and their targets (Herc3, Map3k1, Nedd4, and Cdkn1b/p27), cell signaling (Jag1, Tcf4, and Spry3), DNA-binding transcription factors (c-Maf, MafB, N-Myc, Nfib, Sp1, and Tcf4), chromatin regulation (Ash1l, Med1/PBP, and Kdm5b/Jarid1b/Plu1), synapse formation (Rab11fip2 and Shank2), and FGF/MAPK signaling (c-Maf, Map3k1, and Spry3). The connectivity data for two groups, including Ash1l, Med1/PBP, and Kdm5b/Jarid1b/Plu1, and Cpsf6 and Tnrc6b, are shown in Figure 7, B and C, respectively. Mediator 1/peroxisome proliferator activator receptor−binding protein (Med1/PBP, see Figure 7B) is a coactivator of Gata3 and other Gata DNA-binding transcription factors and gene targeting demonstrated its essential role in lens fiber cell differentiation (Crawford *et al.* 2002). Cleveage and polyadenylation specificity factor 6 (Cpsf6) regulates processing of long noncoding RNAs (Naganuma *et al.* 2012). The RNA recognition motif−containing protein Tnrc6b binds argonaute proteins and mediate miRNA-guided mRNA cleavage and forms the cytoplasmic P bodies (Meister *et al.* 2005; Baillat and Shiekhattar 2009). Ash1l is a histone methyltransferase specific for H3 K4 (Gregory *et al.* 2007). In contrast, Kdm5b/Jarid1b/Plu1 is an H3 K4me3 histone demethylase (Seward *et al.* 2007) that is essential for mouse lens fiber cell differentiation (Albert *et al.* 2013). We conclude that miR-9, miR-137, miR-200c, miR-381, miR-455, miR-495, and miR-543 represent an FGF2-dependent system of multiple miRNAs that target specific genes operating in pathways and processes related to the lens differentiation (via c-Maf, Med1/PBP, N-myc, and Nfat5), miRNA-regulated RNA processing (via Cpsf6 and Tnrc6b) and nuclear/chromatin-based processes (via Med1/PBP, As1l, and Kdm5b/Jarid1b/Plu1).

**Figure 8:**
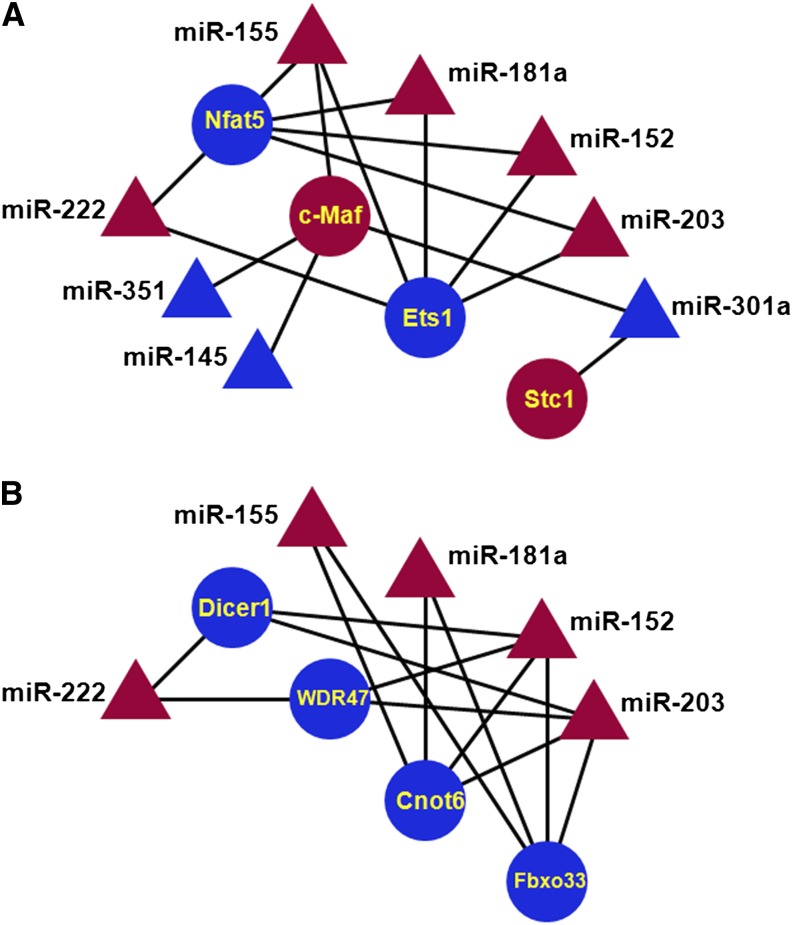
A “late” connectivity network. Ten miRNAs, miR-20b, miR-145, miR-152, miR-155, miR-181a, miR-203, miR-222, miR-301a, miR-324-5p, and miR-351. (A) DNA-binding transcription factors (c-Maf, Ets1, Nfat5/OREBP, and Stc1). (B) mRNA and protein processing (Cnot6, Dicer1, Fbxo33, and Wdr47). miRNA, triangles; mRNAs, circles; up-regulated (red); down-regulated (blue).

Similar analysis of “late” group, including c-Maf, Ets1, N-Myc, Nfat5, and Nfib, yielded 10 miRNAs: miR-20b, miR-145, miR-152, miR-155, miR-181a, miR-203, miR-222, miR-301a, miR-324-5p, and miR-351, with multiple connections. Subsequent analysis yielded 17 target mRNAs, from which we formed four functional groups: DNA-binding transcription factors (c-Maf, Ets1, and Nfat5/OREBP, see Figure 8A), mRNA and protein processing and degradation (Cnot6, Dicer1, Fbxo33, and Wdr47, see Figure 8B), FGF/MAPK signaling (c-Maf, Ets1, and Stc1), and signaling (Aak1, Arhgef12, Nlk, and PPAP2B). In case of c-Maf, an additional connection to miR-155 was shown in T cells (Rodriguez *et al.* 2007; Suzuki *et al.* 2011) and was included in Figure 8A. Endonuclease-exonuclease-phosphatase−type enzyme (Cnot6) functions as mRNA deadenylase, forming the Ccr4-Not complex (Mittal *et al.* 2011; Doidge *et al.* 2012). F-Box protein 33 (Fbxo33) belongs to a family of adaptor proteins that earmark protein substrates for ubiquitination and destruction by the proteasome (Lutz *et al.* 2006). Wdr47/nemitin (neuronal-enriched MAP interacting protein; Wang *et al.* 2012) contains seven WD40-repeats. WD40 proteins form E3 ubiquitin ligase complexes. Interestingly, DDB1, a component of the Cul4 ubiquitin ligase complex, binds WD40 proteins and promotes protein ubiquitination and is essential for lens formation (Cang *et al.* 2006). Additional genes encoding protein processing and degradation part of the GO terms “Ubiquitin mediated proteolysis,” “Ubiquitin-protein ligase activity,” “RNA degradation,” and “Posttransriptional regulation of gene expression” are shown in Figure 4. In summary, the miRNA:RNA connectivity studies demonstrate that several important lens differentiation genes, including c-Maf, Kdm5b/Jarid1b/Plu1, Med1/PBP, Nfat5/OREBP, and N-Myc, are targeted by multiple shared FGF2-regulated miRNAs.

### FGF2-induced miRNAs are key major regulators of cell-cycle arrest

**Figure 9:**
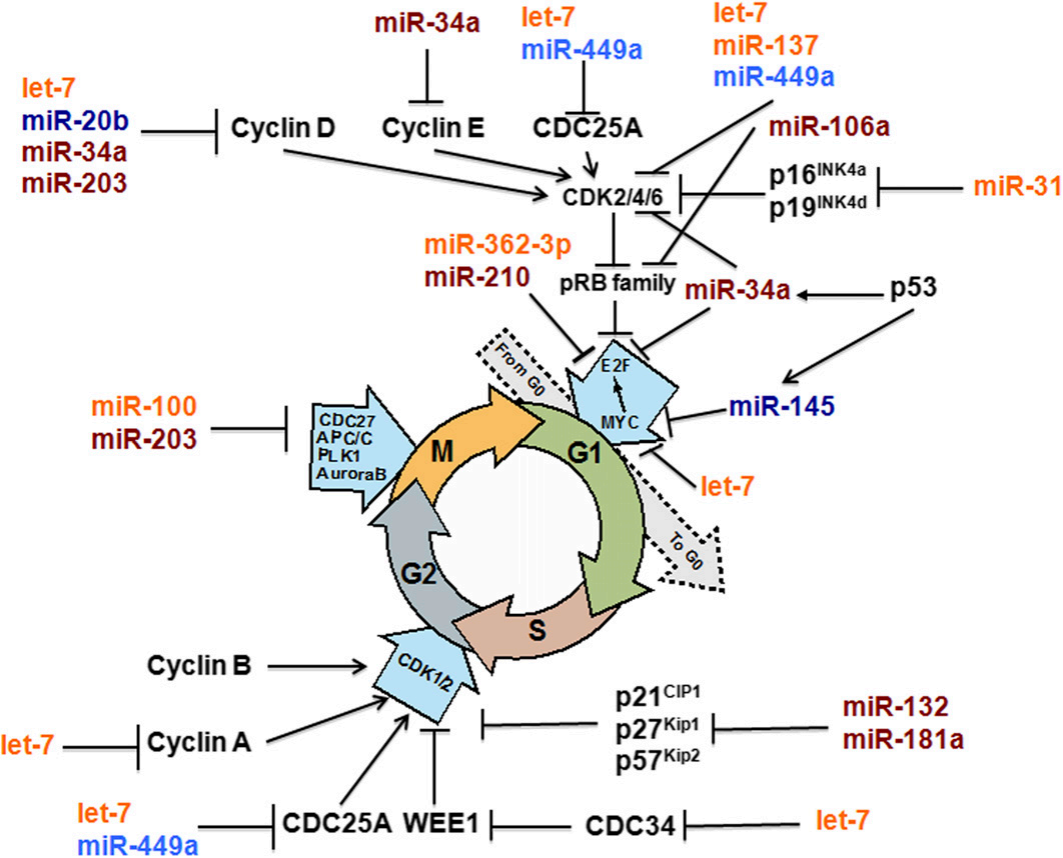
FGF2-induced miRNAs are major regulators of cell cycle arrest. A summary diagram of key regulators of cell-cycle progression/arrest and their FGF2-regulated miRs. This diagram is patterned based on Figure 1, published elsewhere (Bueno and Malumbres 2011). Expression of miR-20b is shown in Figure S2. For miRNA color coding, see legend to Figure 2B.

The initial analysis of aforementioned GO functional categories identified that multiple FGF2-regulated miRNAs target mRNAs encoding well-established regulatory genes of cell-cycle progression (Figure 4). A summary diagram of the cell-cycle−specific regulatory genes and regulatory miRNAs, based on a recent review by Bueno and Malumbres (2011) is shown in Figure 9. We found that 15 (56%) genes in this diagram are regulated by FGF2 through 13 individual miRNA. Cyclins D, Cdc25a, Cdk2/4/6, Myc, Cdc25a, and Plk1, are regulated by at least two distinct miRNAs, including miR-20b (Figure 9). Expression of miR-20b in postnatal day 5 (P5) lens is shown in Figure S2. Expression of miR-20b is most abundant in lens transitional zone, which is in agreement with its presumptive role of controlling cell-cycle exit of the secondary lens fiber cells.

### AP-1 and Ets DNA-binding transcription factors and their regulation by FGF2

Transcription factors from AP-1 and Ets families have been shown to mediate FGF signaling at the level of transcription (Oikawa and Yamada 2003; Buchwalter *et al.* 2004; Turner and Grose 2010). The molecular mechanisms include both transcriptional control and posttranslational modification, including their phosphorylation and SUMOylation (Barolo and Posakony 2002; Tootle and Rebay 2005). Expression of AP-1 genes, including Fosl1, Fosl2, c-Fos, JunB, c-Jun, Fos, Fra, and JunD, and Ets factors, including Ets1, Ets2, Elf1, Etv1/ER81, and Elk1, was evaluated by the arrays. Fra-1/Fosl1, Fra-2/Fosl2, and c-Fos, were strongly induced during the “early” time points. Similarly, the data showed FGF2-dependent induction of Ets1, Ets2, Elf1, and Etv1/ER81 transcripts (File S4).

### Induction and repression of specific BMPs by FGF2

BMP4 and BMP7 have been shown to control various aspects of lens formation (Furuta and Hogan 1998; Faber *et al.* 2002; Sjodal *et al.* 2007; Boswell *et al.* 2008b). Our mRNA:miRNA connectivity analysis (Figure 4) found that multiple components of this pathway are less modulated by FGF2-dependent miRNAs compared with Wnt, MAPK, platelet-derived growth factor, epidermal growth factor, and insulin signaling. Nevertheless, the RNA profiling identified strong up-regulation of Bmp2 and Bmp4 and moderate down-regulation of Bmp7 mRNAs (File S4). Id1, Id2, and Id3 are established BMP signaling-regulated genes (Hollnagel *et al.* 1999; Lopez-Rovira *et al.* 2002; Kowanetz *et al.* 2004). Their expression was induced in the “late” phase of the differentiation. At 4 hr, Id1 and Id3 were already up-regulated whereas expression of Id2 was attenuated. Taken together, these data showed that treatment with FGF2 elicited up-regulation of Bmp4 and Bmp2 as well as Id1/2/3 in “late” stages, indicating that the net effect of this treatment was to activate BMP signaling in this lens explant culture system (File S4).

### Posttranscriptional regulation of c-Maf by FGF2-regulated miRNAs in cultured lens cells

**Figure 10:**
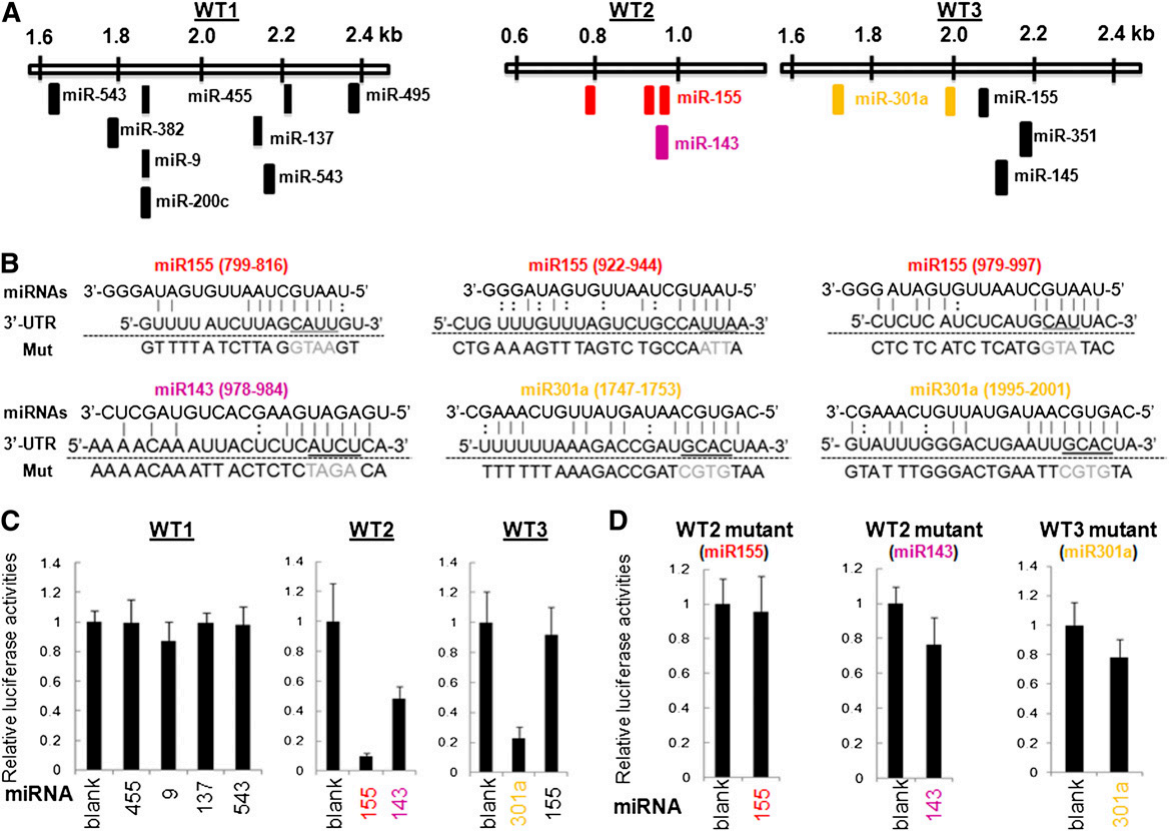
FGF2-regulated miRNAs and posttranscriptional control of c-Maf. (A) Localization of miRNA-binding regions in the mouse c-Maf 3′-UTR (NCBI Reference Sequence NM_001025577.2) and their separation into three shorter regions, WT1 (~0.8 kb), WT2 (~0.2 kb), and WT3 (~0.5 kb). These regions are evolutionary conserved among mammals. (B) Sequence alignment of individual miRNAs:3′-UTR mRNA pairs. Watson-Crick (vertical lines) and wobble (G:U) base pairing are shown. The nucleotides changed in mutated reporter plasmids are highlighted in gray. (C) Luciferase reporter assays using the WT1, WT2, and WT3. (D) Luciferase reporter assays using specific mutants of WT1, WT2, and WT3 (see panel B).

The DNA-binding transcription factors Pax6 and c-Maf are key regulators of lens-specific GRNs that control the crystallin gene machinery (Chauhan *et al.* 2004; Yang *et al.* 2004, 2006; Yang and Cvekl 2005; Xie and Cvekl 2011). Expression of c-Maf, but not of Pax6, is up-regulated in the postmitotic cells of the lens vesicle that give rise to the primary lens fiber cells, raising the possibility that c-Maf is regulated at the level of transcription through FGFs originating from the prospective neuroretina (Lovicu and McAvoy 2005). Subsequent genetic studies supported this model (Zhao *et al.* 2008; Garcia *et al.* 2011; Qu *et al.* 2011). The current data suggest that multiple miRNAs, including miR-9, miR-137, miR-155, miR-301a, miR455, and miR-543 (Figure 7A and Figure 8A), regulate c-Maf expression through its 3′-UTR. In addition, c-Maf 3′-UTR contains a miR-143 target sequence (Figure 10, A and B). Herein, we conducted a series of luciferase reporter gene assays using three wild-type and eight mutated reporters using mouse lens epithelial cell line αTN4-1 (Yang and Cvekl 2005). The c-Maf 3′-UTR was divided into three shorter fragments, WT1 to WT3, as shown in Figure 10A. The predicted 3′-UTR:miRNA pairs for selected six binding sites are shown in Figure 10B. The results of luc reporter assays are summarized in Figure 10, C and D. The data showed that miR-143 and miR-155 down-regulated the WT2 c-Maf 3′-UTR, and miR-301a down-regulated the WT3 c-Maf 3′-UTR (Figure 10C, n = 6, *P* < 0.05). Site-directed mutagenesis of the predicted target sites yielded no statistically significant changes between the wild-type and mutated reporters (Figure 10D).

**Figure 11:**
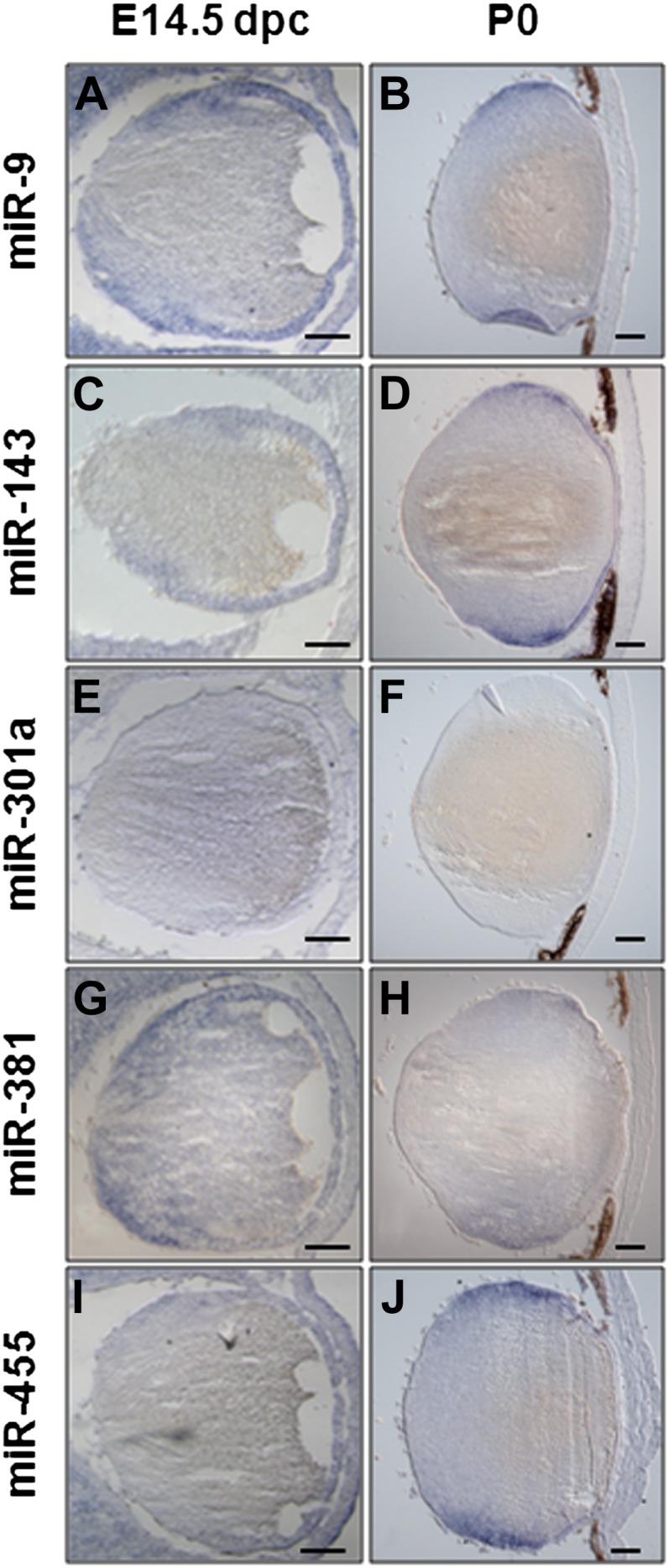
Analysis of miR-9, -143, -301a, -381, and -455 expression pattern during embryonic and postnatal lens development. (A to K′) RNA *in situ* hybridization on frontal eye sections of wild-type mice at different developmental stages (as indicated in the panels) were hybridized with specific probes. At E14.5, the miR-9 (A), -143 (C), -301a (E), -381 (G), and -455 (I) expression domain included the monolayer of lens epithelial cells, the proliferating lens cells, the migrating lens cells, and the differentiating lens cells. Note for miR-301a (E), a weak staining is detectable in these structures at E14.5. At postnatal day P0, the distribution of both miR-9 (B) and miR-143 (D) is largely maintained in all the lens cells previously described for the E14.5 lens, whereas miR-301a (F) is not detected. At this stage both miR-381 (H) and miR-455 (J) expression domains are restricted to the equatorial zone of the lens in proliferating, migrating, and differentiating lens cells.

To determine whether the aforementioned miRNAs identified in rat lens explant system are also expressed during mammalian lens development *in vivo*, we conducted ISH analysis of miR-9, miR-143, miR-155, miR-301a, miR-381, and miR-455 in E14.5 and newborn (P0) lenses. Expression of five miRNAs was shown in the transitional zones of both E14.5 and P0 lenses (Figure 11); however, expression of miR-155 was not established in the mouse lens (data not shown). Taken together, the present data demonstrate that miR-143 and miR-301a are novel regulatory miRNAs for c-Maf expression in mammalian lens.

## Discussion

In this study we identified novel FGF2-regulated miRNA:mRNA GRNs that accompany the process of *in vitro*−induced lens fiber cell differentiation. The identification process included both experimentally determined expression levels of mRNAs (via high density oligonucleotide hybridizations) and miRNAs (via qPCR) followed by *in silico* high-score prediction of inversely correlated mRNA:miRNA pairs, and graphical reconstruction of predicted GRNs. A number of studies have demonstrated computational power of these predictive algorithms (Selbach *et al.* 2008; Guo *et al.* 2010). We predict that several important regulatory genes of lens fiber cell differentiation, including c-Maf, Kdm5b/Jarid1b, Med1/PBP, Nfat5/OREBP, and N-Myc, are connected by multiple shared miRNAs, with four of them, including miR-381, miR-495, miR-382, and miR-543, encoded by a miRNA cluster on rat chromosome 6, a syntenic region with mouse chromosome 12, and human 14q32.2 imprinted regions. Expression of the lens-differentiation factor c-Maf was predicted to be regulated by multiple miRNAs and experimentally validated for three miRNAs, including miR-143, miR-155, and miR-301a, in lens cells. Gene targeting of Dicer1 in differentiating lens demonstrate that miRNAs plays multiple important roles during late stages of lens fiber cell terminal differentiation.

Lens fiber cell differentiation represents an advantageous model system to study how extracellular signals induce cell-cycle exit-coupled terminal differentiation (Lovicu and McAvoy 2005; Griep 2006). Differentiation of rat lens epithelial explants by FGF2 mimics key features of lens fiber cell differentiation (McAvoy and Chamberlain 1989; Zelenka *et al.* 2009; West-Mays *et al.* 2010). In the present study we conducted a time-course analysis of mRNAs and miRNAs expression during the first 24 hr, including four distinct time-points. Lens fiber cell differentiation *in vitro* under the control of FGF2 resulted in a coordinated up- and down-regulation of batteries of genes and noncoding RNAs, including a group of 131 miRNAs that target more than 3000 of individual transcripts. The inverse miRNA:mRNA correlation studies identified as many as 1879 transcripts coregulated by at least two distinct miRNAs, and specific groups of genes modulated by selected groups of miRNAs. These novel FGF2-regulated, miRNA-modulated predicted GRNs are summarized in Figure 6, Figure 7, Figure 8, and Figure 9. The present data show that expression of c-Maf, an essential regulatory factor of lens fiber cell differentiation, is under negative control of multiple miRNAs. The miR-155 has been identified to regulate c-Maf in T cells (Rodriguez *et al.* 2007; Suzuki *et al.* 2011) and was linked to multiple genes within the Ras/MAPK cascade disrupted in chronic myeloid leukemia (Machova Polakova *et al.* 2013). Although we confirmed these data for lens cells, we could not identify expression of miR-155 in mouse E14.5 and P0 lenses. However, it is possible that there are expression differences between mouse and rat lens, and it is also possible that the explant system aberrantly induces its expression.

Our data predict that expression of Med1/PBP, a coactivator of Gata3, is modulated by FGF2-regulated miRNAs. Both Gata3 and Med1/PBP are essential genes of lens formation, namely for lens fiber cell differentiation (Crawford *et al.* 2002; Maeda *et al.* 2009). Both c-Maf and Med1/PBP are predicted to be regulated by similar miRNAs, including miR-137, miR-200c, and miR-495 (Figure 7). In addition, this network of genes includes two chromatin remodeling enzymes, histone methyltransferase Ash1l and its antagonist, the histone demethylase Kdm5b/Jarid1b/Plu1. Kdm5b has been recently shown to be critical for mouse lens fiber cell differentiation (Albert *et al.* 2013).

Earlier lens-specific inactivation of Dicer1 in the prospective lens placode demonstrated that miRNAs play multiple functions during lens formation (Li and Piatigorsky 2009). Here, we used a different Cre-driver, that is expressed much later (difference of ~60 hr) in the lens (Zhao *et al.* 2004), to unravel miRNA roles in the lens differentiation process. Lens-specific inactivation of Dicer1 perturbed the lens fiber cell differentiation process before the E16.5 stage and resulted in the formation of cataract. Compared with the earlier Dicer1 depletion that resulted in reduced expression of β/γ-crystallins (Li and Piatigorsky 2009), the present study did not reveal major decrease in their abundance in the mutated lens. The most likely reason for these findings is more dramatic impact on lens morphogenesis if the inactivation of Dicer1 is initiated in the prospective lens ectoderm compared with the differentiating secondary lens fiber cell. In the abnormal lens fibers, nuclei were not degraded, suggesting that Dicer1/miRNAs participate in lens fiber cell denucleation. The denucleation of lens fiber cells is a hallmark of lens differentiation. Denucleation/karyolysis also occurs in mammalian erythrocytes and skin keratinocytes, albeit through distinct molecular mechanisms (Caceres *et al.* 2010; Wang *et al.* 2010). Our recent studies have shown that ATP-dependent chromatin remodeling enzymes Brg1 and Snf2h and coactivator Ncoa6 are independently required to eliminate lens fiber cell nuclei (He *et al.* 2010; Wang *et al.* 2010). In addition, secondary defects in retinal formation suggest that Dicer1 can play cell nonautonomous roles in retinal development that originate from aberrant lens morphogenesis. Taken together, the present data demonstrate that miRNA-dependent processes are used in the degradation of lens fiber cell nuclei, most likely through genes implicated in clearance of the organelles via autophagy and mitophagy (Brennan *et al.* 2012; Brennan and Giles 2013; Costello *et al.* 2013; Morishita *et al.* 2013).

In addition to the direct role of Dicer1 in lens development demonstrated via conditional gene targeting, our data also suggest that shared FGF2-modulated miRNAs regulate multiple genes involved in miRNA synthesis and function, including Cnot6, Cpsf6, Dicer1, and Tnrc6b (Figure 7 and Figure 8). RNA processing and translational control have been recently shown to be critical for lens differentiation via studies of TDRD7 (Lachke *et al.* 2011) and eIF3 (Choudhuri *et al.* 2013). Down-regulation of Dicer1, predicted by miR-152, miR-203 and miR-222, could be a part of a complex regulation related to the global abundance of miRNAs and their requirements for processes such as cell-cycle exit control and onset of cell differentiation. We conclude the present data support the idea that FGF signaling exerts its role in lens fiber cell differentiation through regulation of genes implicated in miRNA (Cnot6, Dicer1, and Tbrc6b) and general regulation of mRNA processing (Cpsf6).

Although only a few genes within the BMP signaling pathway appear to be regulated via FGF2-regulated miRNAs (Figure 4), the present data show major regulation of three BMPs, including Bmp2, Bmp4, and Bmp7, and their target genes, including Id1, Id2, and Id3, by FGF2 in the rat lens explant system. Interestingly, Bmp4 and Bmp2 were up-regulated as well as Id1, Id2, and Id3 BMP-readout genes. In contrast, expression of Bmp7 was moderately down-regulated in “late” stages of lens explant differentiation. Our data suggest that FGF2-induced BMP2 and BMP4 could work either in the autocrine or paracrine mechanisms in cooperation with FGF to elicit cell-cycle exit as shown in chick models of lens cell-cycle exit-couple differentiation (Boswell *et al.* 2008a,b; Jarrin *et al.* 2012).

Despite of a large number of cell culture models established to study cellular differentiation in response to FGF signaling, RNA expression profiling was so far examined through FGF1 treatment of a cultured rat chondrosarcoma chondrocytic cell line (Dailey *et al.* 2003). The differentiation process was examined at 0, 1, 3, 6, 10, and 24 hr. Similar to the present model, the rat chondrosarcoma/FGF1 study identified that the “early” and “mid” response genes were linked to the initiation and maintenance of growth arrest, and “late” genes identified control chondrocyte differentiation (Dailey *et al.* 2003). In both studies, AP-1 factors, including Fra-1/Fosl1, Fra-2/Fosl2, and c-Fos, were strongly induced during the “early” time-points. In addition, the Ets1 DNA-binding factor and FGF receptor 1 were also induced in both systems. However, in lens explants, Ets2, Elf1, and Etv1/ER81 were also up-regulated in the “early” phase of the differentiation process. In contrast, BMP-targets Id1, Id2, and Id3 were up-regulated in the lens and down-regulated in the chondrocyte system, respectively. In two recent studies investigators also examined FGF signaling using RNA expression profiling (Nieto-Estevez *et al.* 2013; Yang *et al.* 2013). In neurosphere cell cultures, expression of Id3 was also dependent on FGF2 (Nieto-Estevez *et al.* 2013). Inhibition of FGF signaling by FGFR inhibitor SU5402 reduced expression of Bmp7 but did not change expression of Gata3 (Yang *et al.* 2013). These differences show that there are both common and distinct FGF-dependent mechanisms employed by different types of cells.

The connection between FGF signaling and regulation of miRNA is also poorly understood. Inhibition of FGF signaling through SU5402-treated primitive streak regions of chick embryos identified up-regulation of let-7b, miR-9, miR-19b, miR-107, miR-130b, miR-148a, miR-203, and miR-218 and down-regulation of miR-29a and miR-489 (Bobbs *et al.* 2012). In the lens system, miR-29a was up-regulated by FGF2. In contrast, expression of miR-9 and miR-203 was induced by FGF2 in lens, although they were induced via inhibition of FGF receptors in the embryonic chick model (Bobbs *et al.* 2012). Taken together, future studies are needed to identify miRNAs modulated by FGF signaling and their role on processes controlled by this signaling pathway.

The present studies identified a number of FGF-modulated miRNAs encoded by rat chromosome 6q32. This region is syntenic with human miRNA cluster at 14q32.2 and contains at least 61 individual genes (Glazov *et al.* 2008). Loss of heterozygosity in acute lymphoblastic leukemia demonstrates a crucial physiological function of this region (Agueli *et al.* 2010). Three miRNAs from this cluster, including miR-495, miR-543, and miR-381, represent a group of most highly connected miRNAs in this system (Figure 6A) and regulate together multiple genes known to regulate lens fiber cell differentiation, including c-Maf (Figure 7 and Figure 10). The miR-495 and miR-543 are neighbors, and miR-381 is located ~12.7 kb from miR-495. There is a possibility that these and other adjacent miRNAs originate from a single primary transcript similar to the large noncoding transcript named *Mirg* (Seitz *et al.* 2003) that originates nearby, approximately 12 kb toward the telomere. GO analysis of their target genes found links with multiple biological processes, including neurogenesis, embryonic development, transcriptional regulation, and RNA metabolism (Glazov *et al.* 2008). This gross analysis is consistent with our findings of genes regulated by miR-495, miR-543, and miR-381 in lens that belong to these similar categories (Figure 5). These miRNAs were implicated in neuronal differentiation and, given multiple similarities between lens and neuronal differentiation (Frederikse *et al.* 2012), future studies of these miRNAs will shed new light into the miRNA-dependent processes of lens fiber cell and neuronal differentiation.

The mouse genetic studies demonstrate that miRNAs play important roles in lens fiber cell differentiation and degradation of their nuclei. Through the identification of FGF2-modulated batteries of miRNAs, we predicted several GRNs that include DNA-binding transcription factors (c-Maf, N-Myc, Ets1, Nfat5/OREBP, and Nfib), chromatin regulatory factors (Ash1l, Med1/PBP, and Kdm5b/Jarid1b/Plu1), and RNA processing (Cnot6, Cpsf6, Dicer1, and Tnrc6b) with proven or hypothesized roles in lens differentiation. Multiple FGF2-regulated miRNAs with high-connectivity reside in a miRNA-rich cluster on rat chromosome 6q32 (syntenic region of human 14q32.2, mouse 12qF1). The miR-143, miR-155, and miR-301a down-regulated expression of c-Maf evaluated in cultured lens cells through the 3′-UTR luciferase reporter assays. The present data further support the idea that FGF signaling cross-talks with BMP, Notch, and Wnt during lens fiber cell differentiation and demonstrates up-regulation of BMP4 as well as Id1/Id2/Id3 BMP-readout genes in the rat explant cultures. The AP-1 members, including Fosl1, Fosl2, and c-Fos, and Ets members, including Ets1, Ets2, Elf1, and Etv1/ER81, were up-regulated by FGF2, and represent excellent candidates for nuclear factors activated by FGF signaling at the level of gene expression in rat explant system. Collectively, the present studies demonstrate mRNA:miRNA transcriptional responses following activated FGF signaling in lens cell culture system and predicted novel GRNs connected by multiple miRNAs regulating pathway-specific lens regulatory factors.

## Supplementary Material

Supporting Information
