# Supplementary material for: Identification and Characterization of FGF2-Dependent mRNA: microRNA Networks During Lens Fiber Cell Differentiation
Source: G3 (Bethesda). 2013 Oct 18;3(12):2239–55. doi: 10.1534/g3.113.008698 (PMC3852386; doi:10.1534/g3.113.008698)
Supplement: Supporting Information [file supp_3_12_2239__index.html]

Identification and Characterization of FGF2-Dependent mRNA: microRNA Networks During Lens Fiber Cell Differentiation — Supporting Information 

# Identification and Characterization of FGF2-Dependent mRNA: microRNA Networks During Lens Fiber Cell Differentiation

## Supporting Information for Wolf *et al.*, 2013

**Files in this Data Supplement:**

- Supporting Information - Figures S1-S2, Files S1-S4, and Table S1 (PDF, 418 KB)
- Figure S1 - Self organizing maps on duplicate temporal profiles to identify main miRNAs expression profiles. (PDF, 358 KB)
- Figure S2 - ISH miR-20b. (PDF, 320 KB)
- File S1 - Two .xls files, inversely correlated genes. (.zip, 174 KB)
- File S2 - List of genes identified by GO and KEGG (.xlsx, 36 KB)
- File S3 - Two .xls files, connectivity and ranking (.zip, 174 KB)
- File S4 - Expression analysis of Bmp2, Bmp4, Bmp7, Id1, Id2, Id3, Fosl1, Fosl2, c-Fos, Ets1, Ets2, Elf1 and Etv1/ER81 (.xlsx, 42 KB)
- Table S1 - Expression data on 131 microRNAs modulated by FGF2 in rat lens explants (.xlsx, 43 KB)
