# Supplementary material for: Identification and Characterization of FGF2-Dependent mRNA: microRNA Networks During Lens Fiber Cell Differentiation
Source: G3 (Bethesda). 2013 Oct 18;3(12):2239–55. doi: 10.1534/g3.113.008698 (PMC3852386; doi:10.1534/g3.113.008698)
Supplement: Supporting Information [file supp_g3.113.008698_008698SI.pdf]

G3: Genes | Genomes | Genetics

Identification and characterization of FGF2-dependent mRNA:microRNA networks during lens fiber cell differentiation

Louise Wolf[1,2], Chun S. Gao[3], Karen Gueta[4], Qing Xie[1,2], Tiphaine Chevallier[2,5], Nikhil R. Podduturi[6,7], Jian Sun[2], Ivan Conte[8], Peggy S. Zelenka[3], Ruth Ashery-Padan[4], Jiri Zavadil[6,9,*], Ales Cvekl[1,2,*]

The Departments of Genetics[1] and Ophthalmology and Visual Sciences[2], Albert Einstein College of Medicine, Bronx, NY 10461, USA; Laboratory of Molecular and Developmental Biology [3], National Eye Institute (NEI), Bethesda, MD 20892, USA; [4]Sackler School of Medicine and Sagol School of Neuroscience, Tel-Aviv University, 69978 Ramat Aviv, Tel Aviv, Israel; Department of Pathology[6] and New York University Center for Health Informatics and Bioinformatics, New York University Langone Medical Center, New York, NY 10000, USA; Telethon Institute of Genetics and Medicine[8], Via Pietro Castellino 111, I-80131 Naples, Italy

[5]Current address: Institut Cochin, INSERM U1016, Université Paris Descartes, 22 rue Méchain, F-75014 Paris, France.
[7]Current address: The ENCODE Center, Stanford University, Stanford, CA 94305.
[9]Current address: Mechanisms of Carcinogenesis Section, International Agency for Research on Cancer, 69372 Lyon Cedex 08, France.

[*]Corresponding authors: Ales Cvekl, E-mail: ales.cvekl@einstein.yu.edu, and Jiri Zavadil, e-mail: ZavadilJ@iarc.fr

DOI: 10.1534/g3.113.008698

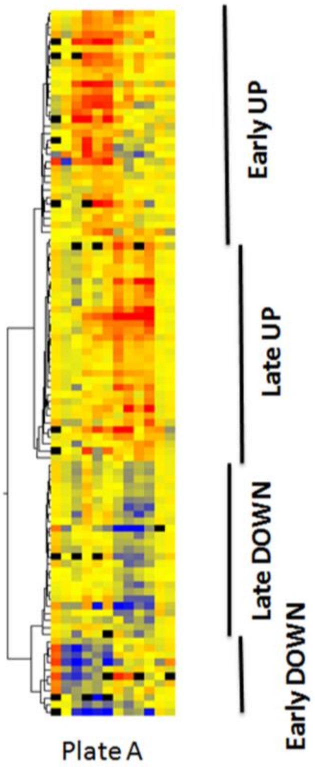

microRNA profile	Regulated (FC > 1.25; 2 time points
"Early" UP	50
"Early" DOWN	16
"Late" UP	36
"Late" Down	29

Figure S1 Self organizing maps on duplicate temporal profiles to identify main miRNAs expression profiles.

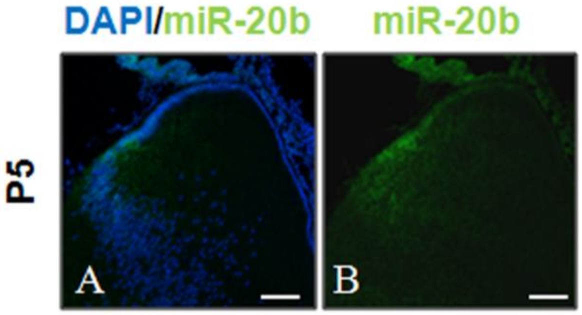

Figure S2 ISH miR-20b. At postnatal day P5, expression of miR-20b (A-B) is detected in the migrating lens cells. Scale bar is 100 µm.

Files S1-S4 and Table S1

Available for download at http://www.g3journal.org/lookup/suppl/doi:10.1534/g3.113.008698/-/DC1

File S1 Two .xls files, inversely correlated genes.

File S2 List of genes identified by GO and KEGG.

File S3 Two .xls files, connectivity and ranking.

File S4 Expression analysis of Bmp2, Bmp4, Bmp7, Id1, Id2, Id3, Fosl1, Fosl2, c-Fos, Ets1, Ets2, Elf1 and Etv1/ER81.

Table S1 Expression data on 131 microRNAs modulated by FGF2 in rat lens explants.
