# Supplementary material for: Identification and Characterization of FGF2-Dependent mRNA: microRNA Networks During Lens Fiber Cell Differentiation
Source: G3 (Bethesda). 2013 Oct 18;3(12):2239–55. doi: 10.1534/g3.113.008698 (PMC3852386; doi:10.1534/g3.113.008698)
Supplement: Supporting Information [file supp_g3.113.008698_FigureS1.pdf]

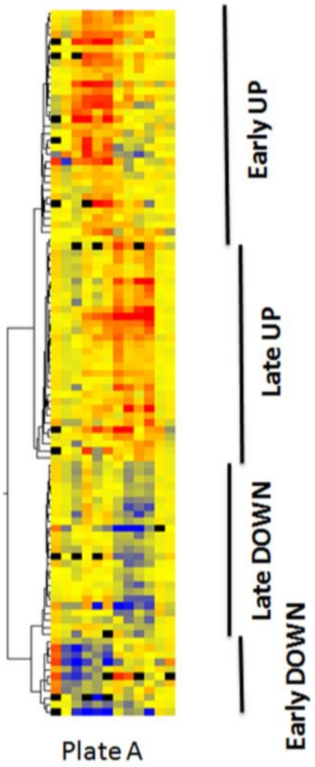

microRNA profile	Regulated (FC > 1.25; 2 time points
“Early” UP	50
“Early” DOWN	16
“Late” UP	36
“Late” Down	29

Figure S1 Self organizing maps on duplicate temporal profiles to identify main miRNAs expression profiles.
