# Supplementary material for: Identification and Characterization of FGF2-Dependent mRNA: microRNA Networks During Lens Fiber Cell Differentiation
Source: G3 (Bethesda). 2013 Oct 18;3(12):2239–55. doi: 10.1534/g3.113.008698 (PMC3852386; doi:10.1534/g3.113.008698)
Supplement: Supporting Information [file supp_g3.113.008698_FigureS2.pdf]

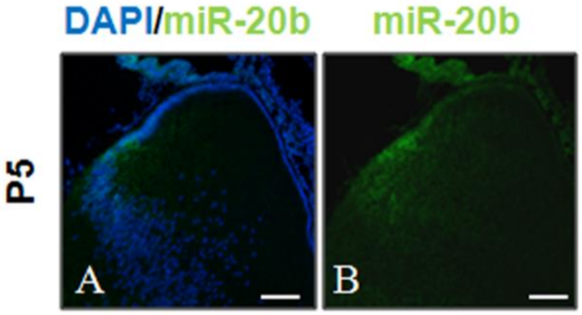

Figure S2 ISH miR-20b. At postnatal day P5, expression of miR-20b (A-B) is detected in the migrating lens cells. Scale bar is 100 µm.
